# Screening Rates and Characteristics of Health Plan Members Who Respond to Screening Reminders

**Published:** 2006-03-15

**Authors:** Jun Zhu, James Davis, Deborah A Taira, Marisa Yamashita

**Affiliations:** Hawaii Medical Service Association, Care Management; Hawaii Medical Service Association and John A. Burns School of Medicine, Honolulu, Hawaii; Hawaii Medical Service Association and John A. Burns School of Medicine, Honolulu, Hawaii; Hawaii Medical Service Association, Honolulu, Hawaii

## Abstract

**Introduction:**

Preventive screening is widely recognized as a key component of cost-effective, high-quality health care. Even so, national screening for cancer, diabetes, and cholesterol falls far short of U.S. Preventive Services Task Force recommendations. Although evidence has shown that reminder programs improve preventive screening rates, this study is one of the first to examine the characteristics of health plan members who respond to screening reminders.

**Methods:**

The study sample included active members of a large health plan in Hawaii who were identified by an algorithm as not having received one or more recommended screenings based on age and sex criteria (2000–2003) for breast cancer (n = 44,331), cervical cancer (n = 73,875), colon cancer (n = 131,860), diabetes (n = 86,216), and cholesterol (n = 54,843).

Statistical analyses were conducted using Cox proportional hazard and logistic regression models. In the proportional hazard models, reminder letters were treated as time-varying exposures. Hazard ratios, or rate ratios, were used to examine the relationship between health plan member and physician characteristics and the likelihood of responding to the reminders. The effects of additional or multiple reminders among health plan members receiving more than one reminder were examined in multivariable regression models.

**Results:**

The impact of health plan member characteristics and number of office visits on the response to reminders varied among the five health-screening types. Health plan members responded better to reminders for diabetes screening than for colon cancer screening. Members sent their second annual reminders were less likely to obtain screening than members sent their first reminder. Members receiving their third (or more) annual reminder were especially recalcitrant.

**Conclusion:**

Our findings suggest that the response to reminders differs according to patient characteristics. In particular, targeted interventions may be needed to encourage screening for younger and healthier members whose response rate to reminders was low. Further research is needed to determine how health plans can best reach members who do not respond to patient reminders.

## Introduction

Preventive screening is widely recognized as a key component of cost-effective, high-quality health care. Even so, national screening for cancer, diabetes, and cholesterol falls far short of U.S. Preventive Services Task Force recommendations (1). Prior evidence suggests that the likelihood of receiving screening is associated with managed care plan activity, including market penetration, type of coverage, and use of gatekeepers (2-5).

In an effort to improve screening rates, many managed care plans send patient and physician reminders for preventive care screening for health plan members who are overdue for screenings based on national guidelines (6-11). Although some studies have shown that these reminders have no impact (12), several meta-analyses have indicated that reminder programs for patients and physicians improve preventive screening rates (13,14). Few studies, however, have focused on understanding which type of patient might benefit from these reminders.

A large health insurer in Hawaii has been providing its members and physicians with screening reminders since 1997. The program aims to provide members with the best opportunity for early detection and successful treatment of breast, cervical, and colon cancer as well as to encourage diabetes and cholesterol screenings. This study monitored members who were overdue for recommended preventive screenings. Members known to have obtained health screenings were not included in the study.

This study sought to understand the relation of health plan member characteristics, physician specialty, and previous health plan member use of medical services to the response to screening reminders for breast cancer, cervical cancer, colon cancer, diabetes, and cholesterol testing. The study also examined the influence of multiple reminders on health plan members overdue for more than one health screening.

## Methods

### Study population

The eligible population included active members of a health maintenance organization who were sent reminder letters about overdue health screenings as indicated by the recommendations of the U.S. Preventive Services Task Force guidelines (1). The guidelines provide criteria based on age, sex, and the frequency of screening. Active members were health plan members who were enrolled with the insurer. The health insurer maintained a registry of health plan members to whom the health screening reminders were mailed. This study used information available in the registry between January 1, 2000, and December 31, 2003. The data used were administrative data, the billing data from insurance claims. The age criteria for screening were 41 to 90 years for breast cancer, 18 to 65 years for cervical cancer, 50 years and older for colon cancer, 45 years and older for diabetes, 35 to 65 years for men for cholesterol, and 45 to 65 years for women for cholesterol. The total numbers of eligible members were 44,331 for breast cancer, 73,875 for cervical cancer, 131,860 (47% men and 53% women) for colon cancer, 86,216 (44% men and 56% women) for diabetes, and 54,843 (58% men and 42% women) for cholesterol. The members consisted approximately of 52% fee for service (FFS), 40% health maintenance organization (HMO), and 8% Medicare plans. Reminder letters were sent every 2 years for breast cancer, every 3 years for cervical cancer, every 5 years for diabetes and cholesterol, and every 1 or 5 years for colon cancer, depending upon the types of previous screening obtained. All active members were sent reminders annually if they were known to have not received the health screenings in accordance with the guidelines. Members were considered overdue even if the registry data were incomplete, as with members new to the insurer, for example. At the beginning of the program, reminders were mailed by the insurer on several dates throughout the year. In 2002, the program began to send birthday card reminders of overdue screenings on the 15th of the members' birth months. Members who were not screened within 1 year were sent an additional reminder on their next birthday. Personal care physicians also received lists of members who had not been screened. Table 1 shows the number of members sent screening reminders by age and sex.

### Health plan member demographics and medical history

The member characteristics examined included the number of screening reminders received (i.e., one, two, or three or more); sex; age classified into nine groups (20 years or younger, 21 to 30 years, 31 to 40 years, 41 to 50 years, 51 to 60 years, 61 to 70 years, 71 to 80 years, 81 to 90 years, and 91 years or older); morbidity level by Adjusted Clinical Groups (ACGs) (12) (on a scale of 0 to 5, measuring the level of illness experienced during the year before the reminders were sent, with 5 representing the greatest illness level); type of coverage (HMO, FFS, or Medicare cost contract plan); the specialty of the primary care physician (including general practice, family practice, and internal medicine, and endocrinologists, cardiologists, obstetricians and gynecologists [OB/GYNs], and "other" specialties); the number of office visits, emergency room visits, and hospitalizations in the past year (classified as no visit or one or more visits); and the calendar months when the reminders were sent.

### Statistical analysis

Statistical analyses were conducted using Cox proportional hazard and logistic regression models. In this study, *additional reminders* refer to more than one reminder for the same type of screening (e.g., breast cancer screening reminders sent in different years); *multiple reminders* refer to the reminders sent for different types of screenings (e.g., breast and colon cancer screening reminders). Reminder letters (additional and multiple) were treated as time-varying exposures in the proportional hazard models, which were used to examine the relationship between member and physician characteristics and the relative response rates to the reminders. The analyses were conducted on the member level by examining the date of a first reminder for a screening type and the date a member received the recommended screening or the date the member's enrollment ended. Members were included in the study for as long as they retained coverage with the insurer, even if they changed health plans. The effects of receiving additional reminders on obtaining a specific health screening in the following year were analyzed using logistic regression. All regression models were adjusted for the island of residence in Hawaii; the number of reminders sent; sex; age group; ACGs; the type of coverage; provider specialty; and the number of office visits, emergency room visits, and hospitalizations.

Screening rates were calculated as the number of members who received screening within the year after reminders were sent divided by the number of members who were sent reminders. All analyses were performed using SAS, version 9.0 (SAS Institute Inc, Cary, NC).

## Results

Of the five health screenings, screening rates were highest for diabetes and lowest for colon cancer (Table 2). The rate of members obtaining the recommended screenings invariably declined with increasing reminder numbers. For example, the response rate for cervical cancer screening was 29.6% after one reminder and 13.0% after three reminders. Screening percentages by sex were consistently higher among women than men but by no more than 6.0% (20.0% versus 16.5% for colon cancer, 29.5% versus 28.9% for diabetes, and 26.3% versus 20.5% for cholesterol testing) (data not shown). 

Screening rates for all five screenings varied by the month the screening reminder was sent (Figure). Screening rates were highest for all health screening reminders sent in June and decreased toward the end of the year. The drop in screening rates from June to December was approximately 20.0% for breast cancer screenings, colon cancer screenings, and diabetes screenings; the drop in screening during those months was 29% for cervical cancer and cholesterol screenings.

**Figure:**
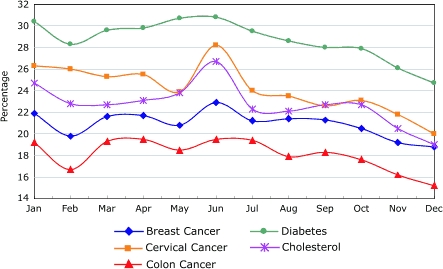
Percentage of members responding to screening reminders by calendar month in which reminders were sent.

**Table:** 

**Month**	**Breast Cancer**	**Cervical Cancer**	**Colon Cancer**	**Diabetes**	**Cholesterol**
Jan	21.9	26.3	19.2	30.4	24.7
Feb	19.8	26	16.7	28.3	22.8
Mar	21.6	25.3	19.3	29.6	22.7
Apr	21.7	25.5	19.5	29.8	23.1
May	20.8	23.9	18.5	30.7	23.8
Jun	22.9	28.2	19.5	30.8	26.7
Jul	21.2	24	19.4	29.5	22.3
Aug	21.4	23.5	17.9	28.6	22.1
Sep	21.3	22.6	18.3	28	22.7
Oct	20.5	23.1	17.6	27.9	22.7
Nov	19.2	21.8	16.2	26.1	20.5
Dec	18.8	20	15.2	24.7	19

In multivariable regression models, rate ratios varied by member characteristics (Table 3). For each of five screening categories, the rate ratios decreased significantly with the number of annual reminders the members received. By contrast, rate ratios varied less by sex; in general, the difference in ratios between men and women was much smaller than the difference in the ratios between age groups. The rate ratios by age groups were generally low for the youngest ages eligible to obtain screening. The rate ratios peaked between the youngest and oldest eligible ages for cancer and diabetes screenings but consistently increased with age for cholesterol screening. For diabetes and cholesterol screening, rate ratios steadily increased with morbidity levels from 0 to 5, whereas cancer rate ratios were highest at morbidity levels of 3 or 4. HMO members had the highest relative rates of breast and cervical cancer screening; Medicare members had the highest relative rates of colon cancer, diabetes, and cholesterol screenings. FFS members consistently had the lowest screening ratios among the three types of coverage.

Table 4 displays the rate ratios by the number of office visits per year, the number of emergency room visits per year, the number of hospitalizations per year, and provider specialties. These data are derived from the year before the members received overdue screening reminders. For breast and cervical cancer screenings, rate ratios varied little with increasing numbers of office visits. For colon cancer, rate ratios increased by 19.0% from 1 to 26 or more office visits. For cholesterol screening, rate ratios for the same comparison increased by 34% but were not statistically significant. Diabetes screening exhibited the strongest trend with office visits; rate ratios increased by 66.0% from 1 to 26 or more office visits. Among the five provider specialties, OB/GYNs had the highest relative screening rates for the three cancer screenings. Only seeing a primary care physician was significantly associated with diabetes screening. Seeing a primary care physician, an endocrinologist, or a cardiologist was significantly associated with obtaining cholesterol screening. Rate ratios among members having an emergency room visit in the past year generally decreased but at most by 11%. Among members having a hospitalization in the past year, only the rate ratio for breast, colon, and cholesterol screenings decreased significantly. 

Members who were sent reminders for multiple overdue screenings were less likely to obtain the recommended screenings than members who were reminded of only one type of overdue screening (Table 5). Members who received reminders for both breast and cervical cancer screenings, for example, had less than half of the screening rates for either cancer compared with members who received a reminder for just one. There were also strong negative associations between receiving a reminder for diabetes screening and also receiving a reminder for cholesterol screening. Members who were sent overdue screening reminders for both screenings were one fourth as likely to obtain the recommended screenings as members sent reminders for only one of the two screenings.

Members who responded to reminders for one type of screening were more likely to obtain other recommended screenings (Table 6). For example, women who were sent reminders for both cervical or breast cancer screenings — and who obtained one of the recommended screenings — were 8 to 10 times as likely to obtain the other cancer screening. Weaker associations were apparent among screening rates for colon, breast, and cervical cancers. Diabetes and cholesterol screenings were strongly associated with each other; screening rates were 20 to 24 times greater among members obtaining one of these health screenings if a screening was obtained for the other.

## Discussion

The impact of patient characteristics and prior use of medical services on the response to reminders varied among the five health-screening types, suggesting that attitudes toward the different types of reminders may differ among health plan members. For example, members responded better to diabetes screening reminders than to colon cancer screening reminders. Members who were sent their second annual reminders were less likely to obtain screening, and those who received their third (or more) reminder were especially recalcitrant. Members who ignore up to three reminders may require other approaches to successfully encourage screening such as phone calls or additional reminders through their personal care physicians.

Although evidence has shown that reminder programs increase preventive screening rates, this study is one of the first to examine the characteristics of health plan members who responded to screening reminders. Age was one of the most consistent factors associated with responding to the reminders. A previous study found that older patients and patients with chronic conditions were less likely to respond to screenings (15). The findings on age are consistent with our results, except that we found older health plan members responded better to cholesterol reminders than younger health plan members. Although women had slightly higher screening rates than men, the rate ratios indicate little difference in the likelihood of response to the screening reminders between men and women.

Morbidity was associated with screening based on an index ranging from ACG level 0, which was considered to be the "healthiest" group, to 5, which was considered to be the most "seriously" ill (12). For the three cancer screenings, the highest screening rates were found at the morbidity levels of 3 and 4, indicating that moderately ill members were more likely to respond to the cancer screening reminders than the healthier or sickest members. Perhaps the healthier members tended to ignore the cancer screening reminders, whereas the sickest members may have addressed their more immediate illnesses rather than their cancer screenings. With diabetes and cholesterol screening, however, screening rates increased directly with morbidity levels. Sicker members (or their physicians) may be more aware of the required health screenings among members repeatedly obtaining health care.

Members more often responded to the screening reminders sent in June or earlier months than to the reminders sent near the end of the year. Low end-of-the-year response rates may be attributable to the year-end holiday season, a season during which people may put off or ignore the recommended screenings. This result may be of practical importance. For some health plan members, reminders sent in addition to their regular birthday reminders may be beneficial in the summer, when members may be more willing to obtain health screenings.  

Members of HMOs and the Medicare plan were more likely to respond to screening reminders than FFS members. One of the major differences between HMOs and Medicare plans compared with FFS plans is that FFS plans do not require a primary care physician. Having a primary care physician may increase screening rates among the HMO and Medicare members. Another explanation could be the difference in coverage for preventive services. FFS members have about 80% of their costs covered by their insurance, whereas HMO and Medicare members (for most screenings) have 100% of their costs covered. The higher member copayment among FFS members may make some members less willing to seek the recommended screenings. In this study, only diabetes screening was significantly affected by the number of health services and especially by the number of office visits, whereas emergency room visits and hospitalization had very little effect on screening rates. The type of physician visited did appear to have selected effects on receiving the recommended screenings. Women who visited an OB/GYN had improved screening rates for breast, cervical, and colon cancer. By contrast, seeing a primary care physician was weakly associated with improved screening rates for diabetes, and seeing an endocrinologist or a cardiologist was associated with improved cholesterol screening rates. 

Members who received reminders for multiple overdue health screenings had reduced screening rates. In particular, women who received a reminder for breast cancer screening and a reminder for cervical cancer screening were much less likely to obtain cervical cancer screening and vice versa. Similarly, members who received a reminder for both diabetes and cholesterol screening had low screening rates for these two health screenings. On the other hand, members who obtained one recommended screening were more likely to obtain others. Women who obtained cervical or breast cancer screening were nearly four times as likely to obtain screening for the other. Similarly, members who obtained a cholesterol or diabetes screening were 14 to 17 times as likely to obtain the other. The strong positive relation between breast and cervical cancer screenings may be because these two cancer screenings are mainly provided by OB/GYNs. The strong positive associations between the diabetes and cholesterol screenings may be attributable to the fact that both screening services are often provided by PCPs. Efforts to improve screening rates might benefit from targeting breast and cervical cancer or cholesterol and diabetes screening as pairs of related screenings.

In this study, several limitations should be considered in interpreting the data. First, the population in this study only consists of the members enrolled with one insurer in Hawaii. The results and conclusions may not generalize to other populations. Second, the analyses are entirely based on administrative data. Any services rendered outside the insurer's system were not recorded. Third, as a practical matter for this study, members were sent reminders if they were considered overdue for recommended screenings. However, some of the members who were considered overdue may have already received the recommended screenings before they enrolled with the insurer. Fourth, family history, household income, and ethnicity were not factored into our statistical models. This information was not available but may have influenced members' health screening behavior. 

Despite these limitations, our findings suggest that response to reminders differs according to patient characteristics. In particular, targeted interventions may be needed to encourage screening for members in the youngest age ranges recommended for screening, which differed by screening category. The younger members who were sent reminder letters had low response rates; the potential to improve their life expectancy and their quality-adjusted life years would be the greatest among the age groups examined in this study. We also found that members who obtained one recommended screening were more likely to obtain others. This finding suggests that sending a single reminder detailing all needed screenings might be the most effective option. Further research is needed to determine how health plans can best reach members who do not respond to patient reminders.

## Figures and Tables

**Table 1:** Number of Health Plan Members Sent Screening Reminders by Age and Sex^a^

**Sex**	**Breast Cancer, Age, y (No.)**	**Cervical Cancer, Age, y (No.)**	**Colon Cancer, Age, y (No.)**	**Diabetes, Age, y (No.)**	**Cholesterol, Age, y (No.)**
Male	NA	NA	≥50 (60,616)	≥45 (36,709)	35-65 (31,298)
Female	41-90 (42,245)	18-65 (71,697)	≥50 (68,398)	≥45 (46,514)	45-65 (22,391)

a NA indicates not applicable.

**Table 2:** Screening Response Rates to Reminders by Number of Reminders Sent and Type of Screening

**No. of Reminders**	**Type of Screening**				

	**Breast Cancer Rate, %**	**Cervical Cancer Rate, %**	**Colon Cancer Rate, %**	**Diabetes Rate, %**	**Cholesterol Rate, %**
1	23.9	29.6	21.5	30.8	25.3
2	19.1	20.0	15.8	26.4	20.4
≥3	12.7	13.0	13.3	23.3	18.5

**Table 3:** Screening Rate Ratios by Health Plan Member Characteristics and Type of Screening^a^

**Member Characteristic**	**Type of Screening^b^**				

	**Breast Cancer Ratio (95% CI)**	**Cervical Cancer Ratio (95% CI)**	**Colon Cancer Ratio (95% CI)**	**Diabetes Ratio (95% CI)**	**Cholesterol Ratio (95% CI)**

**No. of reminders**					

1	1.00	1.00	1.00	1.00	1.00
2	0.61 (0.58-0.64)	0.64 (0.62-0.66)	0.71 (0.69-0.73)	0.95 (0.93-0.98)	0.75 (0.72-0.78)
≥3	0.37 (0.35-0.40)	0.38 (0.37-0.40)	0.52 (0.50-0.54)	0.79 (0.76-0.83)	0.62 (0.60-0.65)

**Sex**					

Male	NA	NA	1.00	1.00	1.00
Female	NA	NA	1.09 (1.07-1.12)	0.97 (0.94-0.99)	1.04 (1.00-1.07)

**Age, y**					

≤20	NA	1.00	NA	NA	NA
21-30	NA	1.96 (1.86-2.06)	NA	NA	NA
31-40	NA	1.63 (1.55-1.71)	NA	NA	1.00
41-50	1.00	1.39 (1.33-1.46)	1.00	1.00	1.43 (1.37-1.49)
51-60	1.15 (1.08-1.22)	1.04 (0.99-1.10)	1.13 (1.08-1.17)	1.22 (1.19-1.25)	1.83 (1.75-1.92)
61-70	1.01 (0.95-1.08)	0.75 (0.70-0.80)	1.16 (1.11-1.20)	1.14 (1.10-1.18)	1.95 (1.82-2.08)
71-80	0.47 (0.42-0.52)	NA	1.10 (1.04-1.15)	0.80 (0.76-0.84)	NA
81-90	0.21 (0.18-0.24)	NA	0.79 (0.74-0.83)	0.66 (0.61-0.72)	NA
≥91	NA	NA	0.48 (0.42-0.55)	0.63 (0.51-0.79)	NA

**Morbidity level^c^**					

0	1.00	1.00	1.00	1.00	1.00
1	1.40 (1.28-1.52)	1.36 (1.30-1.42)	1.41 (1.33-1.49)	1.16 (1.11-1.21)	1.26 (1.20-1.32)
2	1.57 (1.44-1.70)	1.53 (1.46-1.61)	1.63 (1.54-1.72)	1.39 (1.33- 1.45)	1.60 (1.52-1.69)
3	1.80 (1.65-1.97)	1.74 (1.65-1.84)	1.80 (1.70-1.91)	1.63 (1.55- 1.71)	2.06 (1.94- 2.19)
4	1.74 (1.57-1.92)	1.76 (1.64-1.88)	1.75 (1.64-1.86)	1.68 (1.58- 1.78)	1.98 (1.82- 2.16)
5	1.62 (1.45-1.82)	1.66 (1.52-1.81)	1.70 (1.59-1.81)	1.75 (1.62-1.88)	2.38 (2.13-2.66)

**Type of coverage**					

Fee for service	1.00	1.00	1.00	1.00	1.00
Health maintenance organization	1.48 (1.43-1.54)	1.53 (1.49-1.57)	1.47 (1.44-1.51)	1.27 (1.24-1.30)	1.58 (1.53-1.63)
Medicare	1.21 (1.13-1.30)	1.21 (1.00-1.48)	1.54 (1.49-1.59)	1.63 (1.44-1.86)	1.86 (1.31-2.64)

a Analyses were adjusted for the island of residence in Hawaii and for the characteristics listed in Table 4.

b CI indicates confidence interval; NA, not applicable.

c Adjusted Clinical Group (ACG) morbidity levels were determined by Johns Hopkins University School of Hygiene and Public Health (2). The ACG morbidity levels measure the level of overall illness experienced on a scale of 0 to 5, with 5 indicating the most ill.

**Table 4:** Screening Rate Ratios by Medical History and Type of Screening^a^

**Medical History^c^**	**Type of Screening^b^**				

	**Breast Cancer Ratio (95% CI)**	**Cervical Cancer Ratio (95% CI)**	**Colon Cancer Ratio (95% CI)**	**Diabetes Ratio (95% CI)**	**Cholesterol Ratio (95% CI)**

**Office visits in past year**					

0	1.00	1.00	1.00	1.00	1.00
1	1.08 (1.01-1.14)	1.03 (0.99-1.07)	1.07 (1.03-1.10)	1.25 (1.20-1.29)	1.01 (0.92-1.12)
2-5	1.04 (0.99-1.09)	1.02 (0.98-1.06)	1.09 (1.06-1.13)	1.41 (1.37-1.46)	0.97 (0.89-1.05)
6-10	1.01 (0.94-1.07)	0.99 (0.94-1.04)	1.16 (1.12-1.21)	1.64 (1.57-1.71)	1.05 (0.91-1.21)
11-25	1.08 (1.00-1.16)	1.05 (0.98-1.12)	1.19 (1.14-1.24)	1.84 (1.74-1.95)	1.22 (0.97-1.53)
≥26	0.97 (0.83-1.13)	1.06 (0.91-1.23)	1.27 (1.16-1.39)	2.08 (1.78-2.42)	1.35 (0.78-2.34)

**Emergency room visits in past year**					

0	1.00	1.00	1.00	1.00	1.00
≥1	0.89 (0.83-0.95)	0.94 (0.90-0.98)	0.89 (0.86-0.93)	0.97 (0.92-1.02)	1.01 (0.84-1.20)

**Hospitalizations in past year**					

0	1.00	1.00	1.00	1.00	1.00
≥1	0.82 (0.75-0.90)	0.97 (0.91-1.04)	0.85 (0.81-0.90)	1.00 (0.93-1.07)	0.67 (0.49-0.90)

**Provider specialty**					

Primary care	1.06 (1.01-1.11)	0.91 (0.88-0.95)	1.17 (1.14-1.20)	1.06 (1.03-1.09)	1.04 (1.00-1.09)
Endocrinology	1.07 (0.97-1.18)	0.96 (0.88-1.05)	1.17 (1.11-1.24)	1.04 (0.95-1.13)	1.22 (1.06-1.41)
Cardiology	1.01 (0.95-1.08)	0.88 (0.82-0.95)	1.02 (0.99-1.06)	1.02 (0.96-1.07)	1.13 (1.02-1.24)
Obstetrics/gynecology	1.99 (1.90-2.08)	1.84 (1.76-1.91)	1.58 (1.54-1.63)	0.99 (0.96-1.02)	0.98 (0.93-1.03)
Other	1.10 (1.05-1.15)	1.06 (1.02-1.09)	1.20 (1.17-1.22)	0.91 (0.88-0.94)	0.93 (0.89-0.97)

a Analyses were adjusted for the island of residence in Hawaii and for the characteristics listed in Table 3

b CI indicates confidence interval.

c Data are from the year before health plan members received screening reminders.

**Table 5:** Screening Rate Ratios by Multiple Reminders for Overdue Health Screenings Sent to Health Plan Members and by Type of Screening^a^

**Reminders**	**Type of Screening^b^**				

	** Breast Cancer Ratio (95% CI)**	**Cervical Cancer Ratio (95% CI)**	**Colon Cancer Ratio (95% CI)**	**Diabetes Ratio (95% CI)**	**Cholesterol Ratio (95% CI)**
Breast cancer	—	0.22 (0.20-0.23)	0.46 (0.44-0.48)	0.80 (0.76-0.83)	0.85 (0.79-0.91)
Cervical cancer	0.38 (0.37-0.40)	—	0.54 (0.51-0.57)	1.09 (1.05-1.13)	0.97 (0.91-1.02)
Colon cancer	0.49 (0.47-0.51)	0.55 (0.52-0.58)	—	0.88 (0.86-0.90)	1.06 (1.02-1.10)
Diabetes	0.88 (0.83-0.93)	0.76 (0.71-0.81)	0.59 (0.57-0.61)	—	0.23 (0.22-0.25)
Cholesterol	0.97 (0.90-1.05)	0.95 (0.88-1.02)	0.58 (0.55-0.62)	0.19 (0.18-0.20)	—

a Multiple reminders refer to reminders for recommended screenings in addition to the listed screening category (i.e., members receiving a reminder for cervical cancer screening when the analysis outcome is breast cancer screening). Rate ratios measure the screening rates for members who received more than one reminder compared with those who did not. Analyses were adjusted for the island of residence in Hawaii and for the characteristics listed in Tables 3 and 4.

b CI indicates confidence interval.

**Table 6:** Screening Rate Ratios by Multiple Screenings Received and Type of Screening^a^

**Screenings**	**Type of Screening^b^**				

	**Breast Cancer Ratio (CI)**	**Cervical Cancer Ratio (CI)**	**Colon Cancer Ratio (CI)**	**Diabetes Ratio (CI)**	**Cholesterol Ratio (CI)**
Breast cancer	—	10.3 (9.6-11.0)	3.65 (3.44-3.87)	1.32 (1.24-1.40)	1.34 (1.23-1.45)
Cervical cancer	7.89 (7.43-8.37)	—	4.62 (4.31-4.96)	1.08 (1.02-1.14)	1.31 (1.22-1.40)
Colon cancer	2.91 (2.76-3.06)	3.66 (3.44-3.89)	—	1.81 (1.75-1.87)	1.47 (1.40-1.54)
Diabetes	1.57 (1.45-1.69)	1.50 (1.39-1.62)	2.53 (2.41-2.65)	—	23.9 (22.8-25.1)
Cholesterol	1.37 (1.25-1.51)	1.60 (1.46-1.74)	2.06 (1.92-2.21)	19.6 (18.7-20.4)	—

a Multiple screenings refer to screenings other than the listed screening category (i.e., members having received screening for cervical cancer when the analysis outcome is breast cancer screening). Rate ratios measure the screening rates for members who received more than one screening compared with those who did not. Analyses were adjusted for the island of residence in Hawaii and for the characteristics listed in Tables 3 and 4.

b CI indicates confidence interval.
